# Jejunal invagination in an adult caused by inflammatory fibroid polyp: a case report

**DOI:** 10.4076/1757-1626-2-6435

**Published:** 2009-08-10

**Authors:** Gökhan Çipe, Fevzi Celayir, Hakan M Köksal, Sadik Yildirim, Adil Baykan, Havva Elif Öğüt

**Affiliations:** 1 Department of General Surgery Sisli Etfal Research and Training HospitalIstanbul, 81080Turkey; 2 Department of Pathology, Sisli Etfal Research and Training HospitalIstanbul, 81080Turkey

## Abstract

**Introduction:**

Invagination is a rare cause of mechanical intestinal obstruction in adults, but half of their causes are malignant. A diagnosis of invagination in an adult patient strongly suggests presence of a malignant pathology. Moreover some benign conditions may resemble malignant disorders like inflammatory fibroid polyp. Inflammatory fibroid polyps are rare benign lesions of uncertain origin that may occur in various parts of the gastrointestinal tract, with gastric antrum being the most common site, followed by the small intestine.

**Case presentation:**

A 31-year old male patient was admitted to our emergency department few hours after complaints of nausea and vomiting. Abdominal examination revealed distension and clanging intestinal sounds. Computed tomography showed intestinal obstruction without an obvious cause. The patient underwent diagnostic laparotomy. In this process, approximately 10 cm of invaginated mid-jejunal segment was seen. The pathologic segment was resected and end-to-end anastomosis was performed. The patient was discharged without any complication.

**Conclusion:**

Although IFP is seen very rarely in adults, it is one of the probable diagnoses that should be considered in obstructive tumors of the small intestine causing invagination.

## Introduction

Inflammatory fibroid polyp (IFP) is a rare benign condition originating from submucosa [1]. Histologically, a typical IFP constituted by inflammatory proliferation of fibroblasts, blood vessels and dense collagen tissue. Macroscopically, IFP is a solitary, smooth lesion and rarely as a mass lesion, which can be confused with malignant tumors. Erosion and ulceration of mucosal layer can be seen frequently [2-4].

IFP is first described by Vanek as “submucosis granuloma with eosinophilic infiltration” [5]. Afterwards, it termed as hemangiopericytoma, submucosis fibroma, inflammatory pseudotumor and myxoma [6]. Current definition as “IFP” was made by Helwig and Reiner in 1953 [7].

IFP may manifest with a gamut of symptoms depending on localization of lesion. Intestinal IFPs may cause obstruction. Herein, we presented a case of IFP presenting as intestinal invagination in an adult patient.

## Case presentation

A 31-year old Caucasian, Turkish male patient was admitted to our emergency department with the complaints of nausea and vomiting occurring few hours. It was learned that these complaints were repeating after meals for a week, episodically. On admission he looked severely ill and dehydrated. Abdominal examination revealed distension and clanging intestinal sounds. Laboratory investigation showed that leukocytosis (13400/mm^3^), sodium: 134 mmol/L, potassium: 3.2 mmol/L. Plain films of abdomen disclosed multiple intestinal air-fluid levels. Contrast enhanced computed tomography showed intestinal obstruction without an obvious cause.

**Figure 1.**
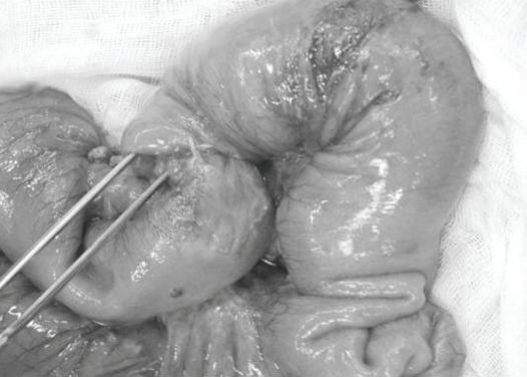
Macroscopic view of invaginated jejunal intestinal segment.

Upon appropriate fluid resuscitation the patient underwent diagnostic laparatomy. In this process, it was seen that an approximately 10-cm of invaginated mid-jejunal segment (Figure 1). The pathologic segment was resected and end-to-end anastomosis was performed. The patient was discharged in a well condition 6 days after the surgery.

**Figure 2.**
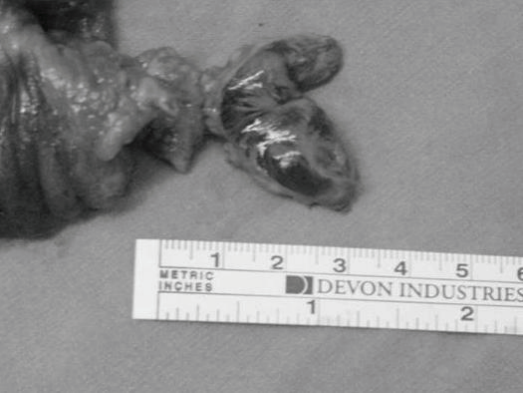
Macroscopic view of inflammatory fibroid polyp with resected intestinal segment.

Macroscopic examination of surgical specimen demonstrated a hard polypoid tumor approximately 3-cm in diameter, originating from submucosa and making ulceration by extending mucosa (Figure 2).

**Figure 3.**
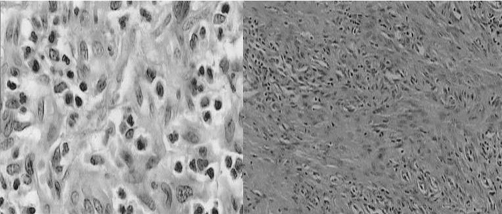
Histopathologic views of inflammatory fibroid polyp.

Histopathologic examination showed proliferated vessel formations in fibrotic stroma, dense inflammatory mononuclear cell infiltration and polypoid tissue with patchy necrotic areas. The connective tissue of polyp was rich in fibroblasts and eosinophils. Vessel congestions were observed at submucosa and mucosa as well. With these findings polypoid lesion was classified as inflammatory fibroid polyp (IFP) (Figure 3).

## Discussion

Intestinal invagination is rarely seen in adults. When diagnosed, it strongly suggests presence of a malignant condition either primary or metastatic origin. Patients with invagination almost inevitably present with complaints of mechanical intestinal obstruction. Definite diagnosis is usually made by a diagnostic laparatomy. Treatment includes medical stabilization of patients’ condition and surgical resection at safe limits [8].

IFP is an extremely rare condition in adults. It may manifest as invagination when located to intestine or as anemia and occult test positivity in the feces when located to stomach. Location of IFP in colon is extremely rare and may present with colicky abdominal pain and bloody feces. At the time of diagnosis most IFPs are in diameter of 3 to 4 cm, however, there is also a report of a case with 12.5 cm in size [9]. Lesion is generally solitary. To date there is only one reported having multicentric localization as named Devon polyposis syndrome [10].

The etiology and pathogenesis of IFP is unknown, but, it could be a consequence of extreme reaction of the body to an intestinal trauma or a localized variant of eosinophilic gastroenteritis given that it has a marked eosinophilic infiltration. [1,3]. However, because an eosinophilic infiltrate can accompany other disorders of gastrointestinal tract eosinophilic infiltration is not a patognomonic finding of IFP.

In conclusion, IFP is one of the probable diagnosis in adults that should be considered in obstructive tumors of the small intestine causing invagination.

## Abbreviation

IFP: Inflammatory fibroid polyp
